# Highly Conductive P-Type MAPbI_3_ Films and Crystals via Sodium Doping

**DOI:** 10.3389/fchem.2020.00754

**Published:** 2020-10-07

**Authors:** Yujiao Li, Chen Li, Huanqin Yu, Beilei Yuan, Fan Xu, Haoming Wei, Bingqiang Cao

**Affiliations:** ^1^School of Physics and Physical Engineering, Qufu Normal University, Qufu, China; ^2^School of Materials Science and Engineering, University of Jinan, Jinan, China

**Keywords:** perovskite, films, doping, optical properties, p-type

## Abstract

To regulate the optical and electrical properties of the crystals and films of the intrinsic methylammonium lead iodide (CH_3_NH_3_PbI_3_), we dope them with sodium (Na) by selecting sodium iodide (NaI) as a dopant source. The highly conductive p-type sodium-doped CH_3_NH_3_PbI_3_ (MAPbI_3_: Na) perovskite single crystals and thin films are successfully grown using the inverse temperature crystallization (ITC) method and antisolvent spin-coating (ASC) method, respectively. With the increase of Na^+^ doping concentration, the grain size of the film increases, the surface becomes smoother, and the crystallinity improves. Hall effect results demonstrate that both the MAPbI_3_: Na thin films and single crystals change their quasi-insulating intrinsic conductivity to a highly conductive p-type conductivity. The room-temperature photoluminescence (PL) peaks of doped MAPbI_3_ films slightly blue shift, while the photocarriers' lifetime becomes longer. The optical fingerprints of the doped levels in MAPbI_3_: Na perovskites can be identified by temperature-dependent PL. Obvious fingerprints of Na-related acceptor (A^0^X) levels in the doped MAPbI_3_: Na were observed at 10 K. These results suggest that sodium doping is an effective way to grow highly conductive p-type MAPbI_3_ perovskites.

## Introduction

Organic–inorganic hybrid halide perovskites have attracted considerable attention in the photoelectric field, due to their low growth cost, long carrier lifetime, low exciton binding energy, and tunable band gap (Xing et al., 2013; Dong et al., 2015; Brenner et al., 2016; Bai et al., 2018; Jena et al., 2019). Perovskite structure has a common ABX_3_ configuration, where A is a monovalent organic or inorganic cation like methylammonium (MA^+^), formamidinium (FA^+^), or Cs^+^; B is a divalent metal ion like Pb^2+^, Sn^2+^, or Ge^2+^; and X is a monovalent anion like Cl^−^, Br^−^, I^−^, or SCN^−^ (Beal et al., 2016). Perovskite materials are widely used for photovoltaics, lasers, photodetectors, light-emitting diodes (LEDs), and thin film transistors (Dai et al., 2014; Dou et al., 2014; Rajagopal et al., 2018; Schulz, 2018). Over the past few years, MAPbI_3_-based perovskite solar cells (PSCs) have made great progress with a current certificated efficiency of 25.2% (NREL). However, perovskite semiconductors have many basic physical properties that are sensitive to the intrinsic defects of the material and to intentional doping, such as bipolar doping, carrier transfer characteristic, etc. However, the level of understanding of these basic semiconductor physics is far below the device fabrication process. For example, doping can control the physical properties of almost all modern semiconductors, which is also the premise used to realize their industrial applications. Many traditional semiconductor materials, like silicon, indium phosphide, and gallium nitride, have achieved controlled bipolar doping of both N type and P type (Wan et al., 2018; Yamada et al., 2019). To change this electronic property, doping technology is widely used in semiconductor-based photovoltaic devices, especially for silicon and CIGS solar cells (Gao et al., 2011; Zhu et al., 2012; Jena et al., 2019). The grain boundaries and crystal surfaces of polycrystalline thin films have inclusion high-density charge traps, which consequently result in the high resistance of perovskite thin films. However, perovskite single crystals have been proven to be able to change their photoelectronic properties by doping due to their high crystallinity, superior optical and electrical properties, and enhanced stability (Jiang et al., 2010; Zhou et al., 2015; Cheng et al., 2019; Gong et al., 2019; Chen et al., 2020).

Because lead halide perovskite is a three-dimensional structure with a high defect tolerance, it can tolerate heterogeneous atom distribution in lattice. Considering the valence distribution in the lattice of hybrid perovskite and the redox resistance of alkali metals, positively charged alkali metal cations are doped into perovskite (Saliba et al., 2016). For example, the incorporation of isovalent Cs^+^ and Rb^+^ into perovskite can improve the power conversion efficiency (PCE) and the stability of PSCs. Cs^+^ doping is favorable for the yellow phase transition (δ-phase) of perovskite, and Zhu et al. found that Cs^+^ partially replaced FA^+^ at position A and can also improve the humidity stability of perovskite due to lattice shrinkage (Li et al., 2016). Saliba et al. added Rb^+^ to the (CsFAMA)Pb(I/Br)_3_ hybrid perovskite, and improved the device stability for 500 h in a nitrogen environment at 85°C (Li et al., 2019a). Adding K^+^ to a perovskite layer can eliminate the hysteresis of the PSC J-V curve (Tang et al., 2017). The addition of Na^+^ in films can prolong carrier life and improve device efficiency (Abdi-Jalebi et al., 2018). But, the detailed physical mechanism is still unclear. Recently, it was proposed to adjust the morphology and optical and electrical properties to improve the photovoltaic performance by replacing Pb with its heterovalent (Slavney et al., 2016; Begum et al., 2017; Jiang et al., 2017; Qiu et al., 2017; Yamada et al., 2017, 2019; Zhang et al., 2017; Ju et al., 2018). But, up to now, there are only very few studies on the basic photoelectric properties of perovskite materials with intentionally doped defects.

Therefore, in order to control the basic physical properties of MAPbI_3_ semiconductors and discuss the influence of alkali metal doping on the conductive and optical properties of MAPbI_3_ perovskite thin films and crystals, we adopt sodium iodide (NaI) as a heterovalent dopant source. Here, we adopt the inverse temperature crystallization (ITC) method and antisolvent spin-coating (ASC) method respectively to grow p-type sodium-doped CH_3_NH_3_PbI_3_ (MAPbI_3_: Na) perovskite single crystals and thin films. With the addition of Na^+^ dopant, the structure of MAPbI_3_ perovskites are still preserved, which is confirmed by the crystal structure characterizations. The effects of Na^+^ doping on perovskite films and crystals are studied with the Hall effect and detailed optical spectrum measurements. Both the MAPbI_3_: Na thin films and single crystals change their quasi-insulating intrinsic conductivity to highly p-type conductivity. The optical fingerprints of MAPbI_3_: Na thin films is investigated in detail by the temperature-dependent PL (TDPL) spectrum. At 10 K, the optical fingerprint of the Na-doping induced impurity levels, like acceptor bound excitons (A^0^X), is found. Therefore, alkali metal doping is an effective way to adjust the semiconductor physical properties of MAPbI_3_ by introducing acceptors, which shows great significance for the development of perovskite electronic devices.

## Experimental Methods

### DFT Simulations

The calculations were performed using density functional theory (DFT) within the projector augmented wave approach (Kresse and Joubert, 1999) and the Perdew–Burke–Ernzerhof (PBE) (Perdew et al., 1996) generalized gradient approximation (GGA), as implemented in the VASP program package (Kresse and Furthmüller, 1996a,b). A 2 × 2 × 2 MAPbI_3_ supercell was adopted for Na-doping calculations with the substitution of a Na atom at Pb site. A plane-wave kinetic energy cutoff of 550 eV and a k-point sampling of 5 × 5 × 5 were used to ensure that all the energy calculations are well-converged to be better than 0.01 me V. Structural optimizations were performed on the pristine and Na-doped MAPbI_3_ with the atomic forces allowed on each atom <0.02 eV Å^−1^.

### Materials

Dimethylformamide (DMF) and dimethylsulphoxide (DMSO) were obtained from Sigma-Aldrich. MAI (99.50%) and PbI_2_ (98.0%) were purchased from Xi'an Paulette Inc. NaI (99.5%) and chlorobenzene were purchased from Aladdin. All the chemicals were used without further purification.

### Preparation of the Perovskite Films

Fluorine-doped SnO_2_ (FTO) glasses were ultrasonically cleaned with washing-up liquid, deionized water, and ethanol for 10 min each. Then, the clean FTO glasses were treated under a plasma cleaner for about 10 min.

Pure MAPbI_3_ solution (1 ml) was prepared by mixing 1 M CH_3_NH_3_I and different concentrations of PbI_2_ (0.99 M, 0.95 M, and 0.9 M) in the mixture of DMF/DMSO (4:1 of volume ratio), which was stirred at room temperature in a glove box. The different ratio of NaI solution (1.0, 5.0, and 10.0%) which dissolved in the mixture of DMF/DMSO (4:1 of volume ratio) was added into the pure MAPbI_3_ solution after standing for 24 h and then stirred for 2 h to gain the precursor solution of MAPbI_3_ doped with Na.

These precursor solutions were spin-coated on FTO glasses in a two-step program at 700 and 4,000 r.p.m. for 3 and 30 s, respectively. During the second step, 150 μL of chlorobenzene was poured on the surface at 25 s before the end. Finally, the substrates were annealed at 100°C for 10 min to gain different ratios of Na-doping MAPbI_3_ perovskite films.

## Results and Discussion

For intrinsic semiconductors, doping with minute amounts of impurity elements will cause a great change in their electronic structure and carrier concentration. In this work, considering the valence distribution in the lattice of hybrid perovskite and the redox resistance of alkali metals, we chose Na atoms as heterovalent dopants. The exact position of such extrinsic cations in the perovskite lattice has been widely discussed. Generally, it is considered that Na^+^ will partially replace A site when it enters the perovskite lattice. But, recently, Li et al. proved that the doping of Na^+^ ions forms the energetically preferred substitution Na_Pb_ through the density functional theory calculations (Li et al., 2019b). Other studies also show that Na^+^ has a small ionic radius with an unfavorable Goldschmidt tolerance factor for occupying the A site of the Pb-I network (Han et al., 2016; Cao et al., 2018). Various studies have shown that the incorporation of alkali cations may occur through different mechanisms, which is still an open question for further study.

**Figure 1:**
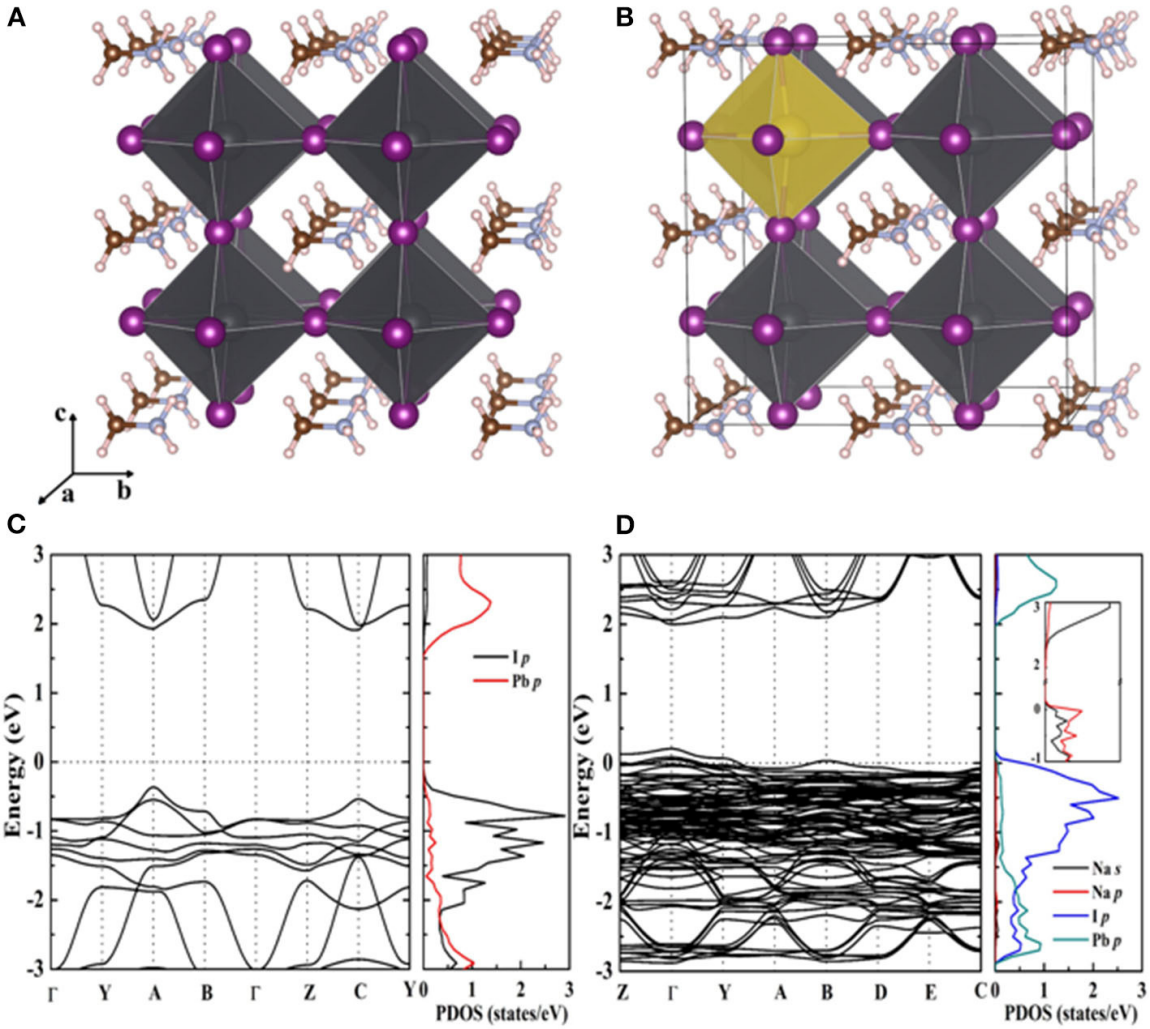
Optimized crystal structures for **(A)** pristine MAPbI_3_ and **(B)** 12.5% Na-doped MAPbI_3_, constructed by the substitution of a Pb atom with a Na atom using a 2 × 2 × 2 supercell. Calculated band structures and partial density of states (DOS) for **(C)** pristine MAPbI_3_ and **(D)** 12.5% Na-doped MAPbI_3_.

Firstly, a 2 × 2 × 2 MAPbI_3_ supercell is adapted to construct a MAPbI_3_: Na model with the cubic perovskite phase (Brivio et al., 2015). A Na dopant atom has taken one Pb atom place in the model, leading to a 12.5% doping concentration. It can be seen that the introduction of Na^+^ does not cause lattice distortion of perovskite compared with the intrinsic MAPbI_3_ from the optimized structure of Figures 1A,B. Before the experiment, the density functional theory (DFT) can calculate the energy band structure and electron state density of MAPbI_3_ (Figure 1C) and MAPbI_3_: Na (Figure 1D), which is helpful to understand the influence of Na doping on the electronic structure of MAPbI_3_. As the figure shows, the partial density of states (PDOS) has peak variation. It is due to the localized impurity band generated by Na that is introduced around the valence band top. And the PDOS indicates that the Pb and I p states' hybridization and Na-s' orbital hybridization together form the doping levels.

To directly prove our theoretical predictions, we prepare MAPbI_3_: Na films and crystals to study the influence of doping on their electronic and optical properties. At first, Na^+^ is selected for heteropolar doping to make monovalent Na replace bivalent Pb in order to obtain p-type perovskite thin films. Among them, the molar ratio of MAI and PbI_2_ is 1: (1, 0.99, 0.95, and 0.9 M) to prepare perovskite solution, which is then mixed with NaI solutions of different concentrations (0:100; 1:99; 5:95; and 10:90). MAPbI_3_ thin films doped with different concentrations of alkali metal Na are prepared using the spinning coating method. Compared with the widely investigated polycrystalline thin films, single crystal perovskites without grain boundaries have better crystallinity and stability, and will be more ideal for investigating the optoelectronic properties. Accordingly, both the MAPbI_3_ crystals and the MAPbI_3_: Na crystals are prepared by the ITC method.

According to the early reports, a 1–2 mm seed crystal is added in the 5 mL transparent solution with a 1.23 mol L^−1^ concentration of MAI/PbI_2_. Then the mother liquor is sealed and maintained at 100°C for 48 h. The seed crystals grow larger to be perfect cubic MAPbI_3_ single crystals with sizes of 9–12 mm. Meanwhile, sodium iodide (NaI) is used for heterovalent doping. We adjust the molar ratio of NaI/PbI_2_ in the mother liquor to 0, 1:99, 5:95, and 10:90. Using the same crystal growth conditions as the intrinsic MAPbI_3_, the seed crystals are sealed in mother liquor at 100°C for 48 h.

**Figure 2:**
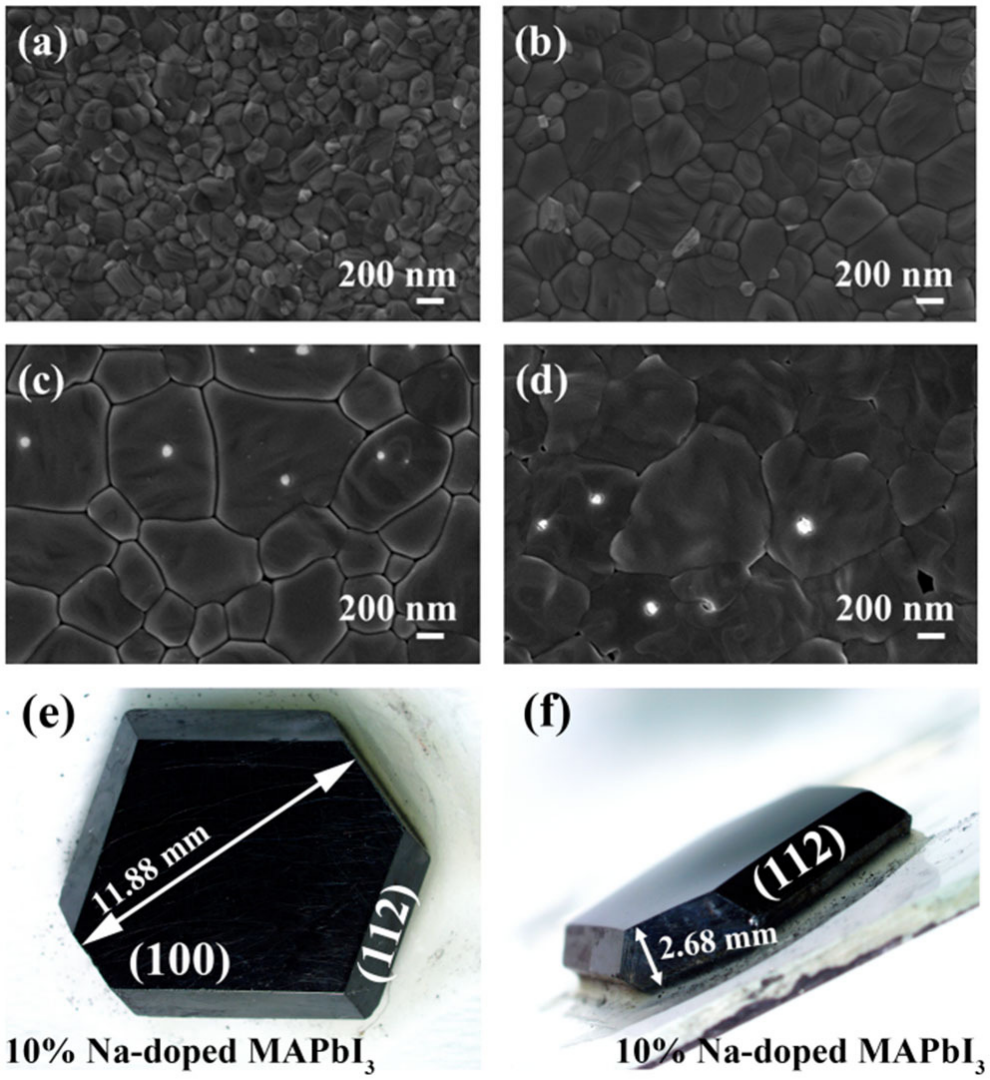
The top-view SEM images of perovskite films grown with precursors of different NaI **(a)** 0%, **(b)** 1%, **(c)** 5% NaI, **(d)** 10%. **(e,f)** optical images of perovskite crystals with different Na doping concentration.

Figures 2a–d show the scanning electron microscopy (SEM) morphologies of perovskite thin films doped with different NaI ratios of 0, 1, 5, and 10%, respectively. With the SEM surface morphology images, we mainly checked the changes of film grain morphology and size with different doping concentrations. All the perovskite films exhibit high coverage. With the gradual increase of doping concentration, the grain size of films increases. So, the addition of Na can promote the film grain growth and the concentration-dependent effect is also significant. Comparative SEM analysis brought out the variations in film evenness and grain size. After NaI is added, the surface of the doped perovskite film is smoother than that of the intrinsic film. However, if the doping concentration is too high, like at 10%, holes appear in the film surface, which will cause current leakage when used for solar cell devices. In the supporting information (Supplementary Figure 1) we have performed elemental analyses to confirm the content of Na dopants. To check the homogeneity of the sodium, we also conduct an EDX mapping test (Supplementary Figure 2). The above data shows that Na^+^ is evenly distributed in the film surface, and the content changes regularly with the controlled precursor. Figures 2e,f display a typical MAPbI_3_ single crystal with the (100) and (112) facets exposed. The MAPbI_3_ single crystals are normally black opaque polyhedrals with a regular cubic morphology.

**Figure 3:**
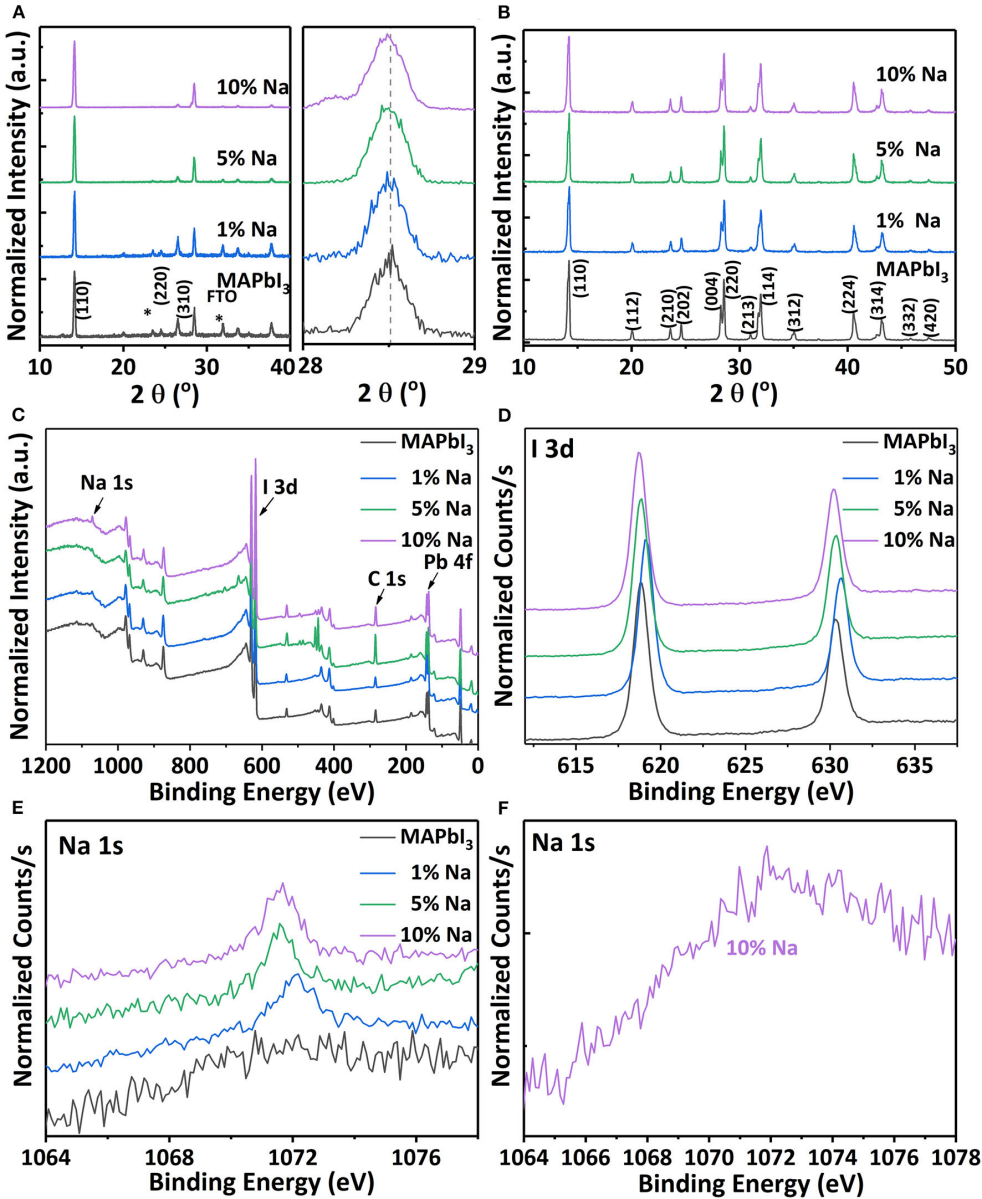
**(A)** The XRD spectra (left) and the local magnification (220) peak (right) of the MAPbI_3_: Na films with different Na/Pb ratios. **(B)** The XRD spectra of perovskite single crystals doped with Na of different concentrations. **(C)** Overview XPS spectra of the controlled MAPbI_3_ and MAPbI_3_: Na films. The surface-sensitive XPS of perovskite films with different doping concentrations for **(D)** I element and **(E)** Na element. **(F)** XPS core-level spectra of the Na 1s peak for the MAPbI_3_ crystal doped with 10% Na^+^.

Figure 3 shows the X-ray diffraction (XRD) patterns of four differently doped MAPbI_3_: Na thin films and crystals. As shown in Figure 3A, the intrinsic MAPbI_3_ films exhibit strong peaks at 14.2 and 28.6°, which are assigned to the (110) and (220) planes of cubic MAPbI_3_ (Abdelhady et al., 2016; Yang et al., 2018). Compared with the intrinsic perovskite samples, the XRD patterns of the Na doped thin film samples remain similar, which implies that the Na is doped into the perovskite structure without any impurity phase. The local magnification (220) peak of the XRD patterns shows a slight peak shift, which may be caused by the substitution of Pb^2+^ with smaller Na^+^ cations to result in lattice contraction. This slight shift is easily overlooked due to the stress tension between the films. And an Na^+^ small peak appears at (220) peak due to 10% high concentration doping. Figure 3B shows the powder XRD patterns of MAPbI_3_: Na crystals, which have more diffraction peaks with Miller indices noted. The diffraction peaks of these samples are consistent with the tetragonal phase of MAPbI_3_ reported in the previous literature (Dang et al., 2015). Na^+^ doping does not introduce any impurity into the MAPbI_3_ single crystal. Therefore, it can be speculated that Na^+^ as dopant is feasible to change the photoelectric properties of intrinsic MAPbI_3_.

X-ray photoelectron spectroscopy (XPS) measurements are conducted on the perovskite samples in order to further verify the existence of the valence states of monovalent alkali metal cations in the perovskite lattice. As shown in Figure 3C, the overview spectra of MAPbI_3_: Na films clearly demonstrate the existence of Na in the MAPbI_3_ and that the oxidation state of Na is +1. We further investigate the influence of Na^+^ incorporation in MAPbI_3_ and MAPbI_3_: Na films using high-resolution XPS analysis. Figure 3D shows that the binding energy of I_3d_ peak shifted with the increase of Na content. The bonding of I^−^ to Pb^2+^ is partially transformed into binding of I^−^ to Na^+^ due to Na^+^ partially replacing Pb^2+^ through doping. The introduction of Na^+^ ions causes abrupt changes in the chemical environment around I^−^, resulting in a bond angle shift. With the increase of Na content, Na^+^ provides more electrons to bond with I^−^, and I^−^ moves toward a low binding energy. The XPS peaks located around 143 and 138 eV, respectively, correspond to the Pb 4f_5/2_ and 4f_7/2_ signals of divalent Pb in Supplementary Figure 3. It is also noticed that the binding energy of Pb 4f shifts as the Na content increases. Figure 3E compares the scanning XPS results of the intrinsic and doped films with different Na doping concentrations. It can be seen that the characteristic peak intensity becomes stronger with the increase of Na^+^ ratio in the precursor solution, indicating that doping is significantly effective. In addition, XPS also confirms the presence of Na in the MAPbI_3_: Na single crystals. The XPS core-level spectra of Na 1S in 10% Na^+^ doped MAPbI_3_: Na crystal is shown in Figure 3F. The peak with binding energy of around 1,072 eV indicates that Na has a + 1 oxidation state in MAPbI_3_: Na crystals. Unlike the 10% Na^+^ doped MAPbI_3_ films, the signal of sodium is relatively weak in doped MAPbI_3_ crystals. Therefore, the thin film samples are more suitable to effectively achieve Na^+^ doping.

The designed Hall effect measurements support the study of the electrical properties of semiconductors. The Hall coefficient can determine the conductivity type of semiconductors, the concentration of carriers, and the mobility of carriers. The MAPbI_3_ perovskite thin films on glass substrates are cut into regular squares with a size of 1 × 1 cm, and gold electrodes of 80 nm are evaporated with a vacuum thermal evaporator at four corners. After the wire bond is completed, a good linear relationship between voltage and current between each point is measured. This indicates that the Au electrode can be used to from good ohm contact with MAPbI_3_: Na.

**Figure 4:**
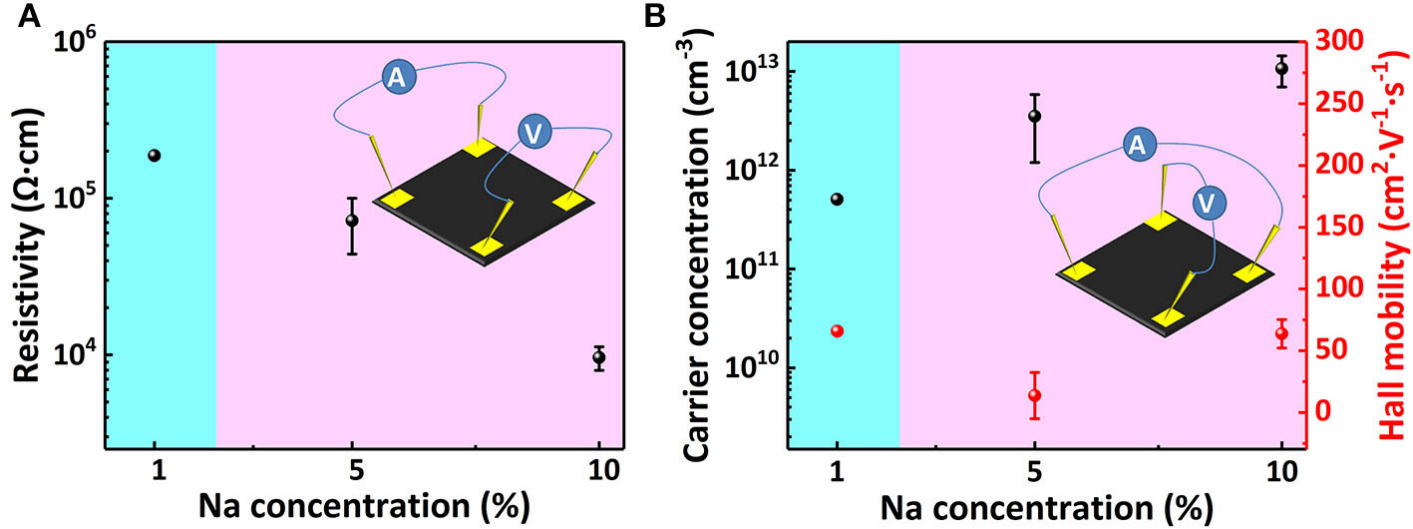
**(A)** Resistivity change, **(B)** carrier concentration, and hall mobility change of Na doped perovskite films at different Na doping concentrations.

The Van Der Pauw method measurement result is shown in Figure 4. Figure 4A shows that, with the increase of Na^+^ concentration, the sheet resistivity decreases by two orders of magnitude, from 10^5^ to 10^3^ Ω cm. This proves that Na^+^ doping significantly improves the MAPbI_3_ perovskite film conductivity, and its conductive type is effectively changed from weak p-type to obvious p-type through doping. Figure 4B shows the changes in carrier concentration and Hall mobility of MAPbI_3_ thin film with increasing doping concentration. After doping, the majority carriers are holes indicating a stable p-type conductivity. With the increase of Na^+^ content, the hole concentration gradually increases to 1,013 cm^−3^, while the hall mobility of the sample decreases slightly. As the doping concentration increases, the experimental error significantly decreases, and the test results tend to be stable and reliable. Unfortunately, the intrinsic polycrystalline perovskite sample resistance is too big for the Hall effect measurement.

MAPbI_3_ single crystals are more reliable for Hall Effect measurement due to their low impurity level and high conductivity. To meet Hall Effect measurement requests for crystal samples, the as-grown MAPbI_3_ crystals are cut and polished. We need to lap the MAPbI_3_: Na crystals until they become ~1 mm thin wafers, and then polish the crystal surfaces. In order to establish an accurate Ohm contact between the test probe and the semiconductor material, Au layers with a thickness of 80 nm are fabricated using the vacuum evaporation method onto the four symmetrical corners of the MAPbI_3_ wafer. The Au layers act as metal contact points. The ohmic contact test results are shown in Supplementary Figure 4. The voltage is almost linear with the current. It is confirmed that the metal and wafer surface form a practicable ohm attachment. The Hall Effect measurement on the perovskite wafers is carried out using the same Van der Pauw method, and its resistivity, carrier concentration, and mobility are summarized in Table 1. We improve measurement precision by averaging the testing values of repeated measurements. The conduction type of intrinsic MAPbI_3_ wafers with a hole carrier concentration of 3.375 × 10^11^ cm^−3^ and conductivity of 3.178 × 10^−6^ Ω^−1^ cm^−1^ is shown to be week p-type. The electronic properties of MAPbI_3_ crystals are insensitive to the concentration of Na^+^ dopant variations. When the Na^+^ doping concentration was 10%, the hole concentration is 1.485 × 10^12^ cm^−3^, and the conductivity increases to 9.699 × 10^−6^ Ω^−1^ cm^−1^. The conduction type is still p-type. The carrier concentration and conductivity show a rising trend with increasing concentration of Na^+^. However, the Hall mobility exhibits an absolutely opposite variation trend with carrier concentration: with increasing concentration of Na^+^, the Hall mobility reduces from 58.79 cm^2^ V^−1^ s^−1^ of intrinsic MAPbI_3_ crystals to 40.77 cm^2^ V^−1^ s^−1^ of 10% Na^+^ doped MAPbI_3_ crystal. Hall effect results demonstrate that both the MAPbI_3_: Na thin films and single crystals change their quasi-insulating intrinsic week p-type conductivity to highly conductive p-type conductivity. On the one hand, due to the increase of Na concentration, more hole carriers are introduced, which increases the conductivity of the sample. On the other hand, combined with the XRD results, the crystallinity of MAPbI_3_: Na films and single crystals have been improved, which is conducive to improving the conductivity.

**Table 1:** Conductivity, charge concentration, and Hall mobility of the crystal wafers doped with Na of different concentrations.

	**μ_*H*_ (cm^2^ v^−1^ s^−1^)**	***p* (cm^**−3**^)**	***σ* (Ω^−1^ cm^−1^)**	**Carrier type**
0% Na	58.79	3.375 × 10^11^	3.178 × 10^−6^	P
10% Na	40.77	1.485 × 10^12^	9.699 × 10^−6^	P

**Figure 5:**
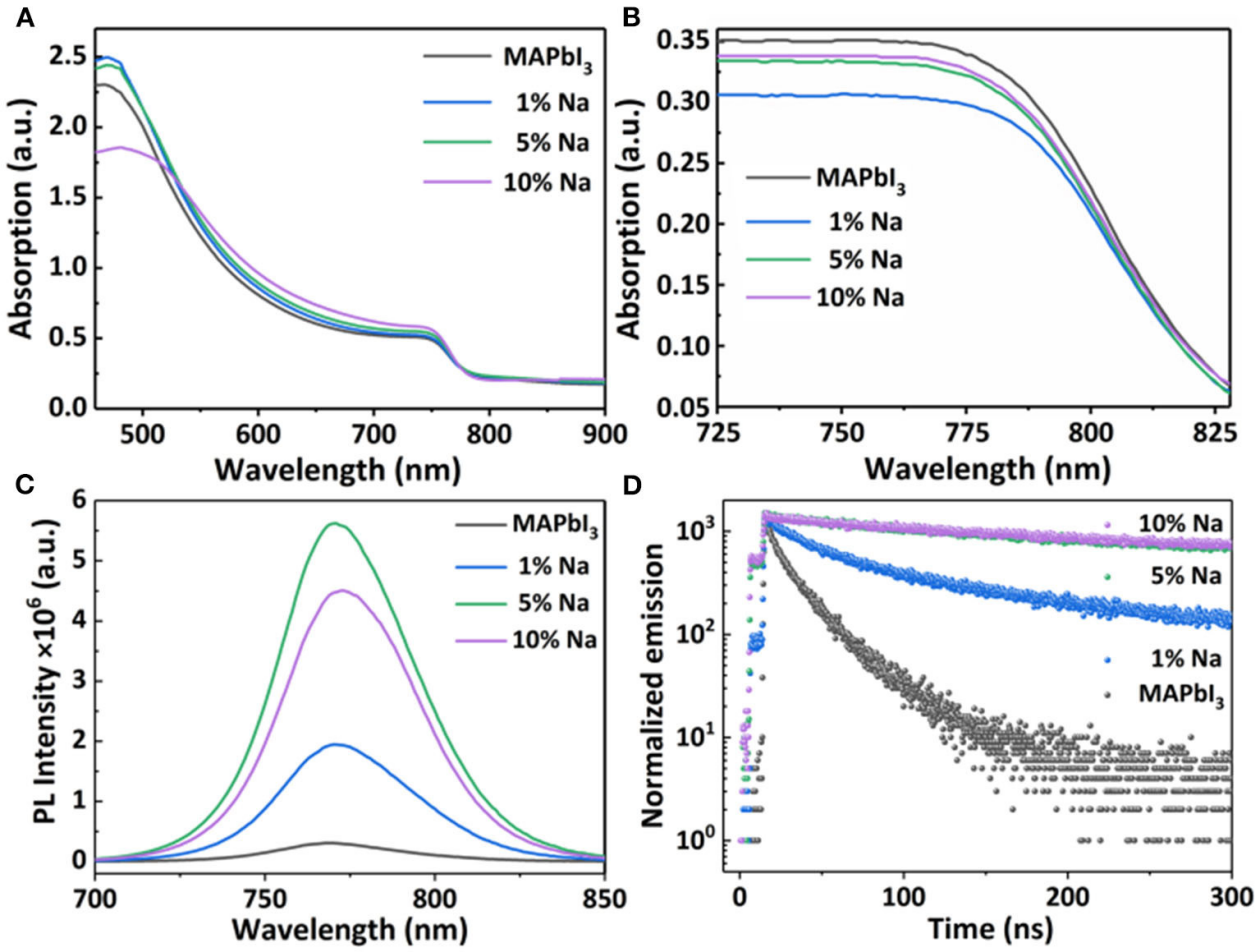
The optical absorption spectrum of **(A)** MAPbI_3_: Na films **(B)** MAPbI_3_: Na single crystals with different doped Na concentrations; **(C,D)** Corresponding photoluminescence and time-resolved photoluminescence spectra of MAPbI_3_: Na films.

In order to further understand the doping mechanism of the electrical properties of an MAPbI_3_ semiconductor, we conducted series optical characterizations on MAPbI_3_. Figure 5 shows the optical absorption spectrum of the Na^+^ doped MAPbI_3_ thin film and crystal. It can be observed from the Figure 5A that the absorption of perovskite films shows differences with different doping concentrations. The absorption starting point is about 780 nm with a corresponding optical band gap of 1.58 eV. The SEM cross section images of the perovskite films with different concentrations are shown in Supplementary Figure 5 and the films' thicknesses are basically close (~300 nm). Under similar thicknesses, the absorption intensity of perovskite films with Na is stronger than that of the pristine perovskite, which can be attributed to the improved crystallinity with Na doping, as proved by SEM and XRD measurements. We used 3D maps of Stylus Profiler to scan a larger area (1,000 ^*^1,000 μm) of the sodium-doped films with different concentrations, and the roughness is roughly similar, as shown in Supplementary Figure 6. Figure 5B shows the optical absorption spectra of Na^+^ doped crystal samples. It was observed that the absorption edges are similar for all MAPbI_3_ single crystals (i.e., pristine and doped). This confirms that the dopant has no influence on the band edge of the perovskite semiconductor.

In order to better understand the effect of Na^+^ doping on the optical properties of MAPbI_3_, we measure the room temperature photoluminescence (PL) spectrum of MAPbI_3_ films and crystals with different Na^+^ doping concentrations. Figure 5C shows the steady-state PL spectra of MAPbI_3_: Na thin films under the excitation of 450 nm light source. When the doping concentration increases from 0 to 5%, the PL intensity gradually increases. This is because the doping of Na^+^ has a grain boundary passivation effect on the perovskite films, which reduces the non-radiative recombination in the perovskite films. At the same time, combined with the above SEM results, with the increase of Na^+^ doping concentration, the grain size gradually increases, the film crystallinity become better, and the PL intensity become stronger. In addition, the Na doping leads the PL peak position, showing a slight shift. Meanwhile, we also measured the PL spectrum of the MAPbI_3_ crystals with different doping concentrations (Supplementary Figure 7). All PL spectra of MAPbI_3_: Na crystals were measured under the excitation of 450 nm xenon lamp. It shows that the PL peak consists of multiple characteristic peaks. This PL peak is attributed to the photon recycling effect, which is due to repeated photon emission and reabsorption processes. Such PL peaks composed by multiple peaks is not observed in thin films, because the photon reabsorption is negligibly small.

In order to further study the optical properties of the doped films, the time-resolved photoluminescence (TRPL) spectrum was measured, as shown in Figure 5D. The TRPL spectra are all well-fitted with a double-exponential attenuation model – I = A_1_exp(-t/t_1_) +A_2_exp(-t/t_2_)—and the fitted lifetimes are summarized in Table 2. As shown in Table 2, the average photocarrier lifetime for MAPbI_3_: Na films are longer than that of the pristine MAPbI_3_ film. Obviously, the addition of alkali metal cations passivates grain boundary defects, reduces the density of trap states, and decreases the non-radiative recombination of photogenerated carriers within the perovskite layer. Therefore, the carrier lifetime of the doped film becomes longer, which corresponds to the higher stable PL intensity. But, the 10% doping concentration is too high, the surface of the perovskite film is rough, and has obvious holes, which leads to the decrease of its PL lifetime.

**Table 2:** The carrier lifetime parameters of Na doped films obtained by fitting the TRPL spectra.

**Sample**	**MAPbI_**3**_**	**MAPbI_**3**_-1%Na**	**MAPbI_**3**_-5%Na**	**MAPbI_**3**_-10%Na**
T1 (ns)	8	27	89	89
T2 (ns)	32	140	1,482	806
τ_average_	16	72	1,180	797

**Figure 6:**
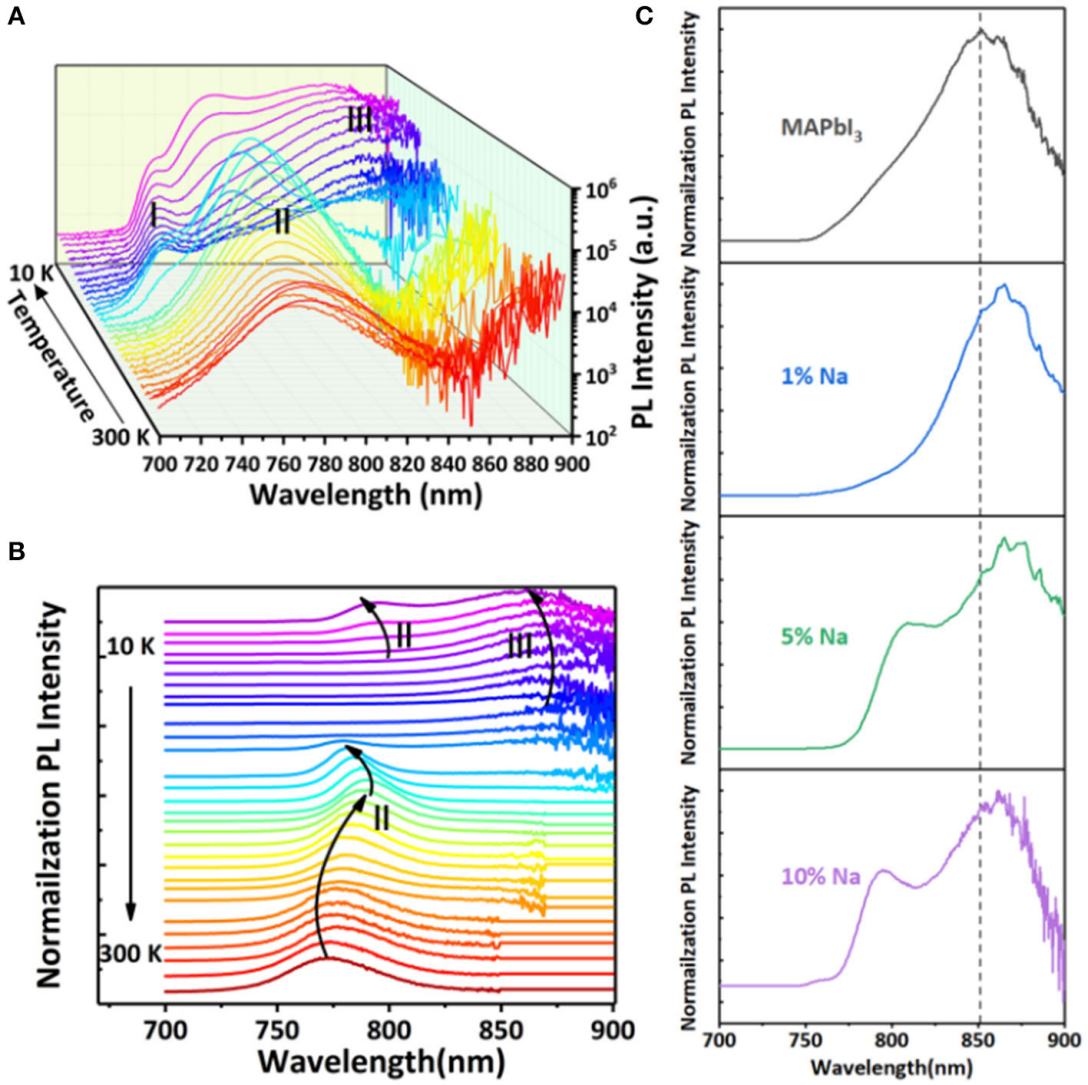
**(A)** Low temperature PL three-dimensional diagram, **(B)** low temperature normalized PL spectrum. **(C)** The steady-state photoluminescence (PL) spectra of different Na doping concentrations at a low temperature of 10 K.

In order to further study the optical fingerprint characteristics of doped thin film, we measure the photoluminescence spectra of MAPbI_3_ thin film doped with 10% Na^+^ as an example in a series of low temperature, as shown in Figure 6A. The normalized PL spectrum with temperature variations is shown in Figure 6B. With temperatures between 130 and 300 K, there is only one obvious emission peak at 780 nm, which is labeled as Peak II. New emission peaks in the PL spectrum appear when the temperature is gradually reduced. From 120 to 10 K, a high energy peak, called Peak I, appears at 740 nm, while another low energy peak, called Peak III, appears at 862 nm. Peak I and III only appear below 120 K, and the peaks' intensity increases significantly with the decrease of temperature. With the decrease of temperature, Peak I appears redshift continuously. On the contrary, the Peak III position appears blueshift. The dependence of Peak II position on temperature is different from that of Peak I and III. The Peak II position redshifts by 15 nm as the temperature drops from 300 to 160 K. And a sudden inversion of the trend of Peak II is observed below 160 K. Between 160 and 120 K, a blue shift by 10 nm of Peak II occurs. Moreover, when the temperature is further reduced to the 120–10 K range, Peak II redshifts again by 14 nm. In summary, the moving track of Peak II's position shifts with temperature decline is similarly S-shaped.

Most often, the temperature-dependence change of the band gap can affect the position of free exciton (FE) peaks, and its change trend has a good consistency. So, Peak I can be considered as a low temperature free exciton transition. According to the energy position of Peak II, we can consider it as it springs from near-bandgap free carrier recombination (FC). Peak II appears as an S-shaped shift with temperature decline from 300 to 10 K. It also illustrates that Peak II is caused by the recombination of free carriers near the edge of the bandgap.

When the temperature decreases to 160 K, MAPbI_3_ thin films undergo a phase transition, which has been extensively studied in the literature (Fang et al., 2015; Frost and Walsh, 2016). To determine the physical origin of Peak III, we compare the spectra of MAPbI_3_ at 10 K with different Na^+^ concentrations, as shown in Figure 6C. The results show that with the increase of Na^+^ doping concentration, Peak III becomes relatively stronger. Combined with Hall data analysis, Peak III is derived from the binding exciton recombination, and the recombination center of the binding exciton is transformed from the intrinsic defect to the acceptor energy level provided by Na^+^, namely the acceptor bound exciton (A^0^X).

## Conclusions

In summary, we successfully doped the monovalent alkali metal Na^+^ ions into the MAPbI_3_ perovskite single crystals and thin films, and the highly conductive p-type MAPbI_3_: Na crystals and thin films are obtained by controlling the Na doping concentration. With the increase of Na^+^ doping concentration, the grain size of the film increases, the surface becomes smoother. And the crystallinity increases. Doping can also passivate the polycrystalline MAPbI_3_ film defects and therefore increase photocarrier lifetime. With the increase of Na doping concentration, hole carrier concentration and p-type conductivity both increases. Three characteristic peaks of MAPbI_3_: Na thin film are found in low temperature PL spectrum. Through the detailed analysis of TDPL spectrum, significant fingerprints of Na-related acceptor (A^0^X) is found at 10 K in doped MAPbI_3_: Na. This indicates that Na doping can partially occupy the Pb site and introduce stable acceptors in MAPbI_3_. This study demonstrates that alkali metals can be successfully doped into MAPbI_3_ perovskite materials, providing new evidence for tuning their optical and electronic properties, which is particularly important for the design of P-N-junction based perovskite optoelectronic devices, such as conventional diodes and solar cells.

## Data Availability Statement

All datasets generated for this study are included in the article/Supplementary Material.

## Author Contributions

BC devised the project and proof outline. YL, CL, HY, BY, FX, and HW synthesized the single crystals and conducted all the characterizations. All authors contributed to manuscript revision and read and approved the submitted version.

## Conflict of Interest

The authors declare that the research was conducted in the absence of any commercial or financial relationships that could be construed as a potential conflict of interest. The reviewer JD declared a past co-authorship with one of the authors BC to the handling Editor.
