# Supplementary material for: Highly Conductive P-Type MAPbI3 Films and Crystals via Sodium Doping
Source: Front Chem. 2020 Oct 7;8:754. doi: 10.3389/fchem.2020.00754 (PMC7575732; doi:10.3389/fchem.2020.00754)
Supplement: Supplementary file 1 [file Data_Sheet_1.PDF]

Supporting Information

Highly conductive p-type $MAPbI_3$ films and crystals via sodium doping

Yujiao Li [a], Chen Li [b], Huanqin Yu [b], Beilei Yuan [b], Fan Xu [a], Haoming Wei [a], Bingqiang Cao [a,b,]*

[a] *School of Physics and Physical Engineering, Qufu Normal University, 273165, Qufu, China*

[b] *School of Materials Science and Engineering, University of Jinan, 250022, Jinan, China*

[*] *Corresponding author*

Email caobq@qfnu.edu.cn

(a)

element	Atomic number	net value	quality [%]	Normalized quality[%]	atom [%]	abs.error [%]	rel.error [%]
Iodine	53	21293	43.30	45.57	30.60	1.51	3.48
Carbon	6	2996	2.62	2.76	19.59	0.48	18.26
Tin	50	14750	22.37	23.54	16.90	0.79	3.54
Nitrogen	7	1587	2.04	2.15	13.05	0.43	21.27
Oxygen	8	2037	1.77	1.87	9.94	0.36	20.27
Lead	82	19514	22.92	24.12	9.92	0.87	3.78
Sodium	11	0	0	0	0	0	3.88
		**total: 95.03**		**100**	**100**		

(b)

element	Atomic number	net value	quality [%]	Normalized quality[%]	atom [%]	abs.error [%]	rel.error [%]
Iodine	53	18162	36.91	40.41	26.52	1.30	3.51
Carbon	6	2981	2.72	2.97	20.62	0.50	18.25
Tin	50	18763	28.16	30.83	21.64	0.98	3.49
Nitrogen	7	961	1.29	1.41	8.37	0.32	24.85
Oxygen	8	2695	2.46	2.69	14.00	0.46	18.72
Lead	82	17097	19.77	21.65	8.70	0.75	3.81
Sodium	11	48	0.04	0.04	0.15	0.01	18.42
		**total: 91.34**		**100**	**100**		

(c)

element	Atomic number	net value	quality [%]	Normalized quality[%]	atom [%]	abs.error [%]	rel.error [%]
Iodine	53	19286	38.76	42.21	26.89	1.36	3.50
Carbon	6	2989	2.65	2.88	19.39	0.48	18.26
Tin	50	18582	27.59	30.05	20.47	0.96	3.49
Nitrogen	7	1272	1.63	1.77	10.23	0.37	22.75
Oxygen	8	3033	2.66	2.89	14.63	0.48	18.20
Lead	82	15977	18.40	20.03	7.82	0.71	3.83
Sodium	11	192	0.15	0.16	0.57	0.04	28.12
		**total: 91.83**		**100**	**100**		

(d)

element	Atomic number	net value	quality [%]	Normalized quality[%]	atom [%]	abs.error [%]	rel.error [%]
Iodine	53	19935	38.85	44.00	28.57	1.36	3.51
Carbon	6	3020	2.49	2.82	19.33	0.45	18.28
Tin	50	18544	26.24	29.71	20.63	0.92	3.50
Nitrogen	7	1176	1.39	1.58	9.29	0.33	23.46
Oxygen	8	2881	2.33	2.64	13.60	0.43	18.52
Lead	82	15789	16.74	18.96	7.54	0.64	3.85
Sodium	11	333	0.26	0.29	1.05	0.05	19.41
		**total: 88.30**		**100**	**100**		

Figure S1. The elemental analyses of perovskite films with different NaI (a) 0 %, (b) 1 %, (c) 5 %, (d) 10 % doping concentration.

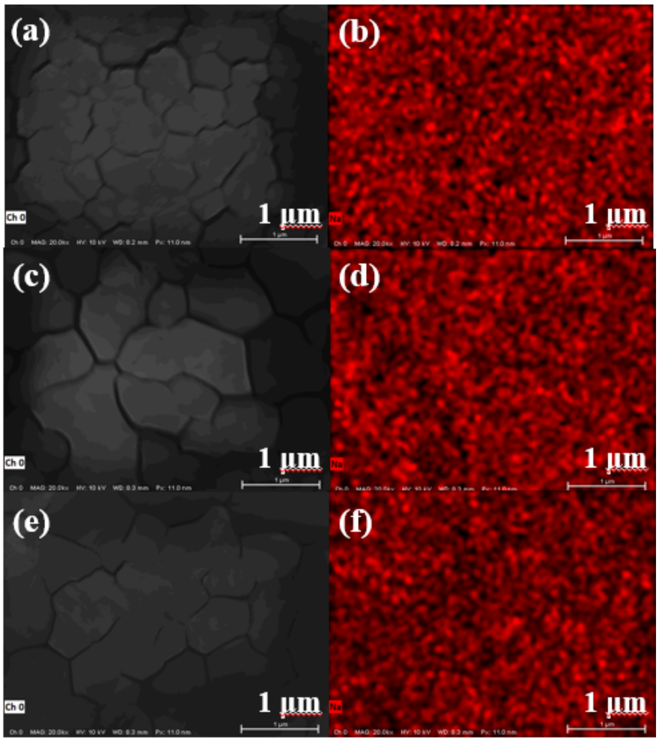

Figure S2. The SEM images and corresponding EDX mapping of perovskite films with different doped NaI concentration. (a-b) 1 %, (c-d) 5 %, (e-f) 10 %.

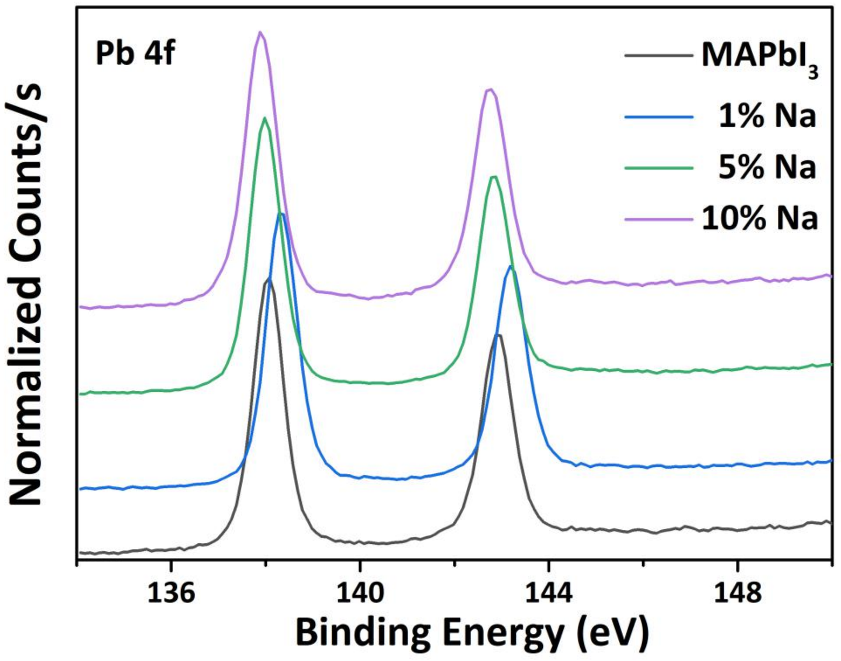

Figure S3. The surface-sensitive XPS of perovskite films with different doping concentrations for Pb element.

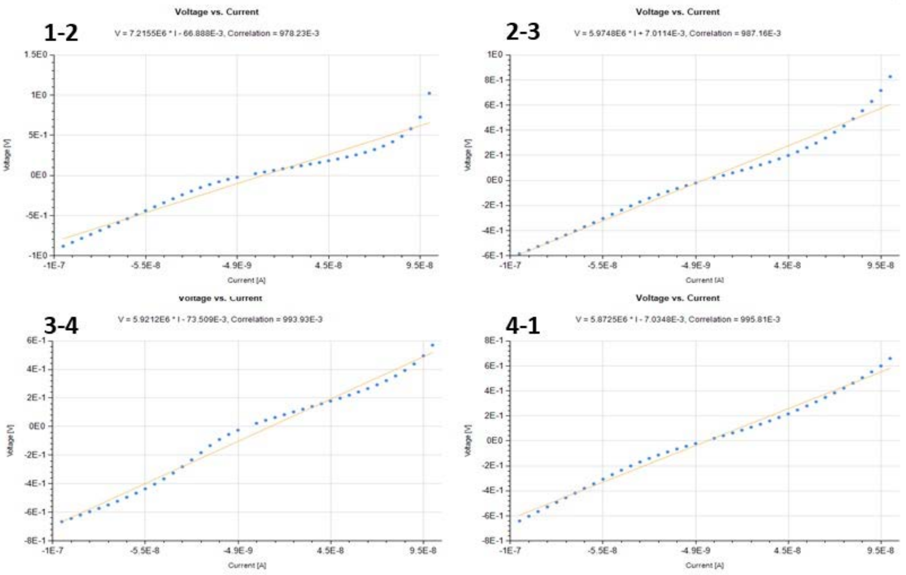

Figure S4. The current-voltage properties to be used for contact check.

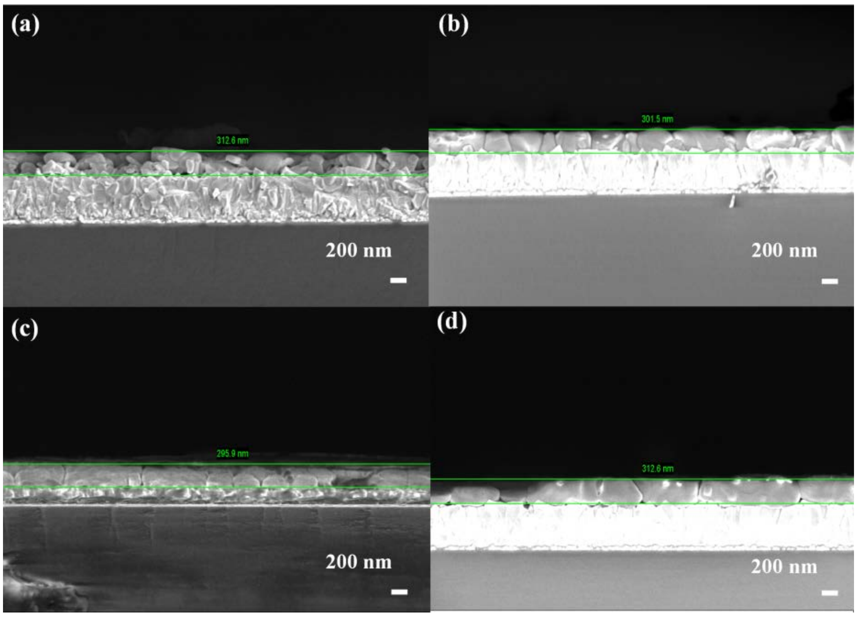

Figure S5. The SEM cross sections of perovskite films with different concentration. (a) 0 %, (b) 1 %, (c) 5 %, (d) 10 %.

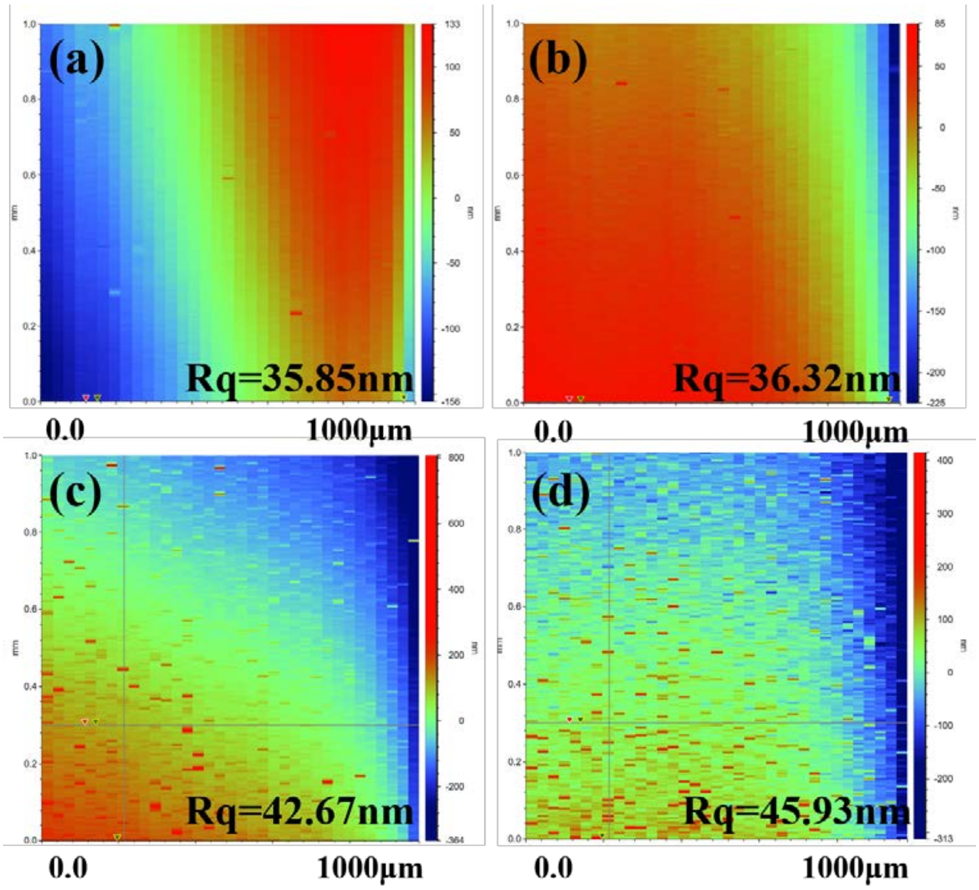

Figure S6. The 3D maps of Stylus Profiler of perovskite films a) without, or with b) 1%, c) 5%, and d) 10% NaI doping. (The surface of the substrate is rough, there is a degree of warpage, resulting in a tilt of the swept plane. Rq is obtained after surface flattening.)

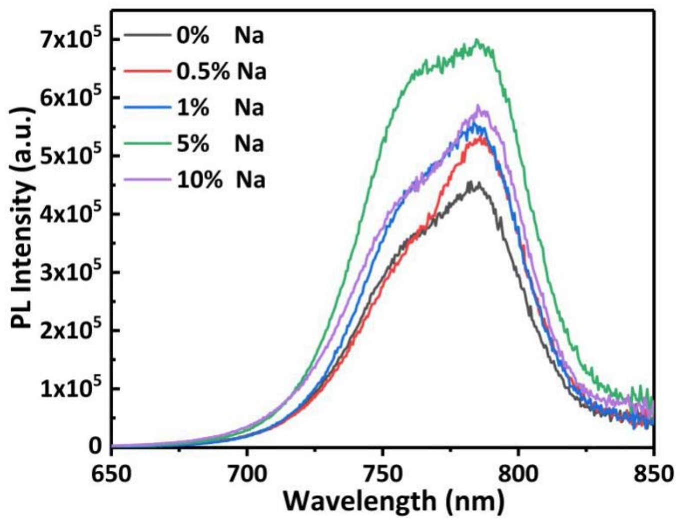

Figure S7. Steady state PL spectrum of the pristine single crystal and Na^{+} doped $MAPbI_3$ crystals.
